# RNU (*Foxn1*^RNU^-Nude) Rats Demonstrate an Improved Ability to Regenerate Muscle in a Volumetric Muscle Injury Compared to Sprague Dawley Rats

**DOI:** 10.3390/bioengineering8010012

**Published:** 2021-01-15

**Authors:** Michael J. McClure, Lucas C. Olson, David J. Cohen, Yen Chen Huang, Shirley Zhang, Tri Nguyen, Barbara D. Boyan, Zvi Schwartz

**Affiliations:** 1 Department of Biomedical Engineering, College of Engineering, Virginia Commonwealth University, Richmond, VA 23284, USA; olsonlc@vcu.edu (L.C.O.); djcohen@vcu.edu (D.J.C.); bunny.day2014@gmail.com (S.Z.); tmnguyen2@mymail.vcu.edu (T.N.); bboyan@vcu.edu (B.D.B.); zschwartz@vcu.edu (Z.S.); 2 MTF Biologics, Edison, NJ 08837, USA; yen_chen_huang@mtf.org; 3 Wallace H. Coulter Department of Biomedical Engineering, College of Engineering, Georgia Institute of Technology, Atlanta, GA 30332, USA; 4 Department of Periodontics, School of Dentistry, University of Texas Health Science Center at San Antonio, San Antonio, TX 78229, USA

**Keywords:** muscle, regeneration, RNU, decellularized tissue scaffold, *Foxn1*, nude rat

## Abstract

Products developed for skeletal muscle regeneration frequently incorporate allogeneic and xenogeneic materials to elicit a regenerative response to heal skeletal muscle wounds. To avoid graft rejection in preclinical studies, immunodeficient rodents are used. Whether the immunodeficiency alters the host response to the material in skeletal muscle has not been studied. In this study, we hypothesized that an allogeneic acellular skeletal muscle grafts implanted in an immunodeficient rat (RNU, *Foxn1*-deficient) would exhibit better new muscle fiber formation compared to grafts implanted in immunocompetent Sprague Dawley (SD) rats. Decellularized SD skeletal muscle matrix (DMM) was implanted in the gastrocnemius (N = 8 rats/group). 56 days after surgery, animal gait was examined and animals were euthanized. Muscle force was assessed and fiber number as well as immune cell infiltrate was measured by histomorphometry and immunohistochemistry. Animal gait and percent recovery of muscle force were unchanged in both groups, but newly regenerated muscle fibers increased in RNU rats. Macrophage staining for CD68 was higher in RNU rats than in SD rats. These data show differences in muscle regeneration between animal models using the same biomaterial treatment, but these differences could not be ascribed to the immune response. Overall, our data provide awareness that more studies are needed to understand how host responses to biomaterials differ based on the animal model used.

## 1. Introduction

Skeletal muscle injuries occur once every thirty-five seconds [1] and elicit a host of responses that result in either full regeneration or fibrosis, depending on the severity of the injury. Severe muscle damage like volumetric muscle loss (VML), fails to repair, causing irrecoverable functional losses and producing prolonged pain around the injury site [2]. A muscle stem cell population (satellite cells) regulates the process of muscle repair and regeneration, and dictates muscle’s regenerative capacity. These cells reside underneath the basal lamina of muscle fibers in a quiescent state, and are activated in response to fiber damage. In cases of acute muscle trauma, shear loss of the motor units, including satellite cells, overwhelms the muscle’s inherent capacity to regenerate, precluding the possibility of healing [3].

Regenerative medicine strategies offer an ability to grow new muscle and replenish satellite cell numbers in cases where severe damage and fibrosis have occurred. Biologic and synthetic materials are commonly developed and used as scaffold materials that either deliver autologous or allogeneic cells or are implanted cell-free and take advantage of the body’s own regenerative and reparative processes. Thus, use of animal models to test behavior of those materials becomes a critical step in understanding how a material responds to injury.

In skeletal muscle studies, Sprague Dawley rats and *Foxn1*^RNU^ (RNU) are two common models used to identify material response. Sprague Dawley rats are a single outbred strain with an intact immune system that has been well characterized in regenerative medicine studies. In contrast, RNU rats, which have a compromised immune system, are used in cases that introduce xenogeneic products into the animal as a means to avoid tissue and cell transplant rejection. RNU rats were established by the National Institutes for Health using a mixture of eight different inbred rat strains, knocking out the RNU allele on chromosome 10 [4]. This transgenic rat produces a T-cell deficiency and shows depleted cell populations in the thymus of peripheral lymphoid organs [5,6,7].

The immune response is critical for normal muscle healing [8], and prior studies suggest its relevance to improved repair in nude rodent models [9,10,11,12]. Studies using immunodeficient nude mice showed improved wound repair with scar-free healing in skin compared to immunocompetent mice [11,12], but whether this is also the case for muscle is not known. RNU rats lacking *Foxn1* are of particular interest for assessing the potential contribution of the immune response to tissue regeneration. It is expressed in skeletal muscle and is involved in early limb development [13], yet reports on the interrelationship between *Foxn1* deficiency and skeletal muscle regeneration are lacking.

Upon injury, neutrophils and macrophages arrive to initiate a pro-inflammatory response. Establishing this pro-inflammatory environment in the injury enables macrophages to prepare the wound site by phagocytosing cell and matrix debris and prompting satellite cells to activate and proliferate [14]. This macrophage response is also closely linked with T-cell response to injury [15]. Nude animals retain CD4 and CD8+ T cells, but at lower levels when compared to normal wild type animals [16], contributing to a communication loss between the innate (macrophage) and adaptive (T-cell) immune systems [17,18].

Muscle tissue pathology is closely linked to macrophage polarization following injury [19], indicating that if T-cell communication is lost, it could drastically alter the microenvironment for regeneration. Indeed, there is a growing body of evidence from healing and regenerative models that suggests the adaptive immune system participates in biomaterial integration [20]. Hotchkiss et al. demonstrated that titanium implants with specific surface properties polarized T cells toward a Th2, pro-wound healing phenotype, leading to faster resolution of inflammation, and when macrophages were ablated, so was the Th2 response [21].

Soft tissue injuries like those in muscle alter the immune response to create an environment that favors fibrosis [22]. In these types of injuries, the wound environment is mostly dictated by the first subpopulation of phagocytic M1 macrophages, while the second subpopulation of M2 macrophages are suppressed, shifting the balance toward fibrosis [23]. Pulmonary arterial hypertension studies in RNU rats showed increased CD68+ macrophage staining in the lung compared to Sprague Dawley rats, suggesting that activated T cells suppressed macrophage migration and supported the idea that macrophage and T-cell regulation needs to be further explored especially in regenerative models [24]. More, increased macrophage migration may also resolve inflammation more quickly. Regulation of an imbalance in macrophage phenotype is therefore a critical feature to consider when assessing the products to be used in cases of severe muscle trauma, especially in animals that disrupt normal communication between T cells and macrophages, preemptively dissuading prolonged inflammatory mediators that could increase fibrosis.

Previously, we developed a decellularized muscle matrix (DMM) in collaboration with MTF Biologics, and tested its ability to regenerate muscle in a VML model using immunocompetent Sprague Dawley rats [25]. Our model was further developed using a 1 cm × 1 cm and 1.5 cm × 1 cm defect in the gastrocnemius to determine whether the size of the defect changed the healing response. When we compared the two models we determined that a smaller 1 cm × 1 cm defect was unable to significantly reduce force output, whereas a larger 1.5 cm × 1 cm defect reduced the force compared to sham animals. We next assessed the graft performance in both models using histology, morphometry, and immunohistochemistry and determined that DMM supported de novo fiber formation, vasculogenesis, satellite cells, and immature acetylcholine receptors within the graft area.

Whether altering the immune response to DMM would also change the host response is unclear. To better understand whether regeneration is affected by immunodeficiency, we compared the effect of immunodeficient RNU rats to immunocompetent Sprague Dawley rats on VML wound healing. As stated, RNU animals are often used to prevent immune rejection and reports indicate that T-cell deficiency in these animals suppresses inflammation in skin wounds, suggesting that muscle may also be affected. Furthermore, T-cell deficient animals expressed a different composition of macrophage subtypes, which could contribute to altered regeneration [10,26]. In this study, we hypothesized that repair of a VML defect with an allogeneic DMM would improve histologic and functional evidence of muscle regeneration in RNU rats when compared to immunocompetent Sprague Dawley rats.

## 2. Materials and Methods

### 2.1. Decellularized Muscle Preparation

All animals were studies were performed in accordance with approved VCU protocols (IACUC #AD10000675). Male Sprague Dawley rats weighing 250–300 g (Envigo, Huntingdon, UK) were euthanized using CO_2_. Gastrocnemius muscles were isolated bilaterally frozen at −80 °C, and shipped to MTF Biologics (Edison, NJ, USA) to be decellularized. Decellularization was performed via a proprietary method composed of multiple saline, detergent, and disinfection soaks using American Association of Tissue Banking and Food and Drug Administration approved protocols developed by MTF. The rat tissue was processed aseptically and without any terminal sterilization. Frozen decellularized muscle matrices (DMM) were shipped back to Virginia Commonwealth University (VCU) and were kept frozen at −80 °C until surgery.

### 2.2. Volumetric Muscle Loss Surgery 

Nineteen male Sprague-Dawley and nineteen male RNU rats (250–300 g) were obtained from Envigo (Huntingdon, UK) and were divided into three groups (N = 8 rats/group for sham and DMM surgeries and N = 3/group for empty defect surgeries). All rats were given ad libitum access to standard pellet and water and provided environmental enrichment. Animals were housed individually. All surgical procedures were performed under an approved protocol at VCU (IACUC #AD10000675) as previously described [25]. Briefly, rats were anesthetized using 4% isoflurane/400 mL/minute O_2_ and prepped for surgery. Rats were transferred to the operating table, and anesthesia was continued at 1–3% isoflurane in O_2_. An oblique anterolateral incision extending from the patella to the calcaneus was made. After an incision in the biceps femoris muscle to expose the gastrocnemius was made, the left lateral gastrocnemius muscle was carefully separated posteriorly off the superficial flexor muscle toward the gastrocnemius origin in the lateral condyle. A 1.5 cm × 1 cm defect was cut in the lateral gastrocnemius, taking care to preserve the sural and tibial nerve. At the end of the surgery, biceps femoris was sutured closed using 5–0 nylon and skin was stapled closed thereafter.

Sham surgeries were performed as described except when the left lateral gastrocnemius was exposed, no incision was made to create a defect. Instead, biceps femoris and skin were closed without a defect (Sham-S and Sham-A, N = 8). Empty defect surgeries were performed as described and left untreated, closing the biceps femoris and skin following tissue harvest (Empty-A and Empty-S, N = 3). Rats that received DMM were sutured into muscle defects, using a modified Kessler technique and taking care to orient the anisotropic features in the direction of the muscle fibers (DMM-S and DMM-A, N = 8). All Sham, Empty, and DMM rats survived and were euthanized at 8 weeks.

### 2.3. Gait Analysis 

Animal gait was assessed at 14 and 56 days using multiple outcome measures to compare the effect of DMM in RNU and Sprague Dawley rats. The experimental setup was adapted from the design by Yu et al. [27] and Chaniary et al. [28]. In this study, animals were randomly chosen and videotaped while spontaneously walking inside a Plexiglas chamber toward a darkened goal box with food. Animals were videotaped and assessed for gait as previously described [25].

Animals were videotaped while spontaneously walking inside a Plexiglas chamber toward a darkened goal box with food. The Plexiglas chamber was a rectangular tube measuring 6 × 6 × 36 inches connected to a darkened box measuring 9 × 9 × 9 inches. Underneath the tube, a grid pattern marked 1 cm apart was used to determine paw placement and hind limb spread. This grid pattern was visualized using a mirror positioned at 45° beneath the chamber. Animals were placed within the tube and the door to the tube was closed and secured. Rats were encouraged to move using food placed in the dark box at the other end of the tube. Videos were taken continuously until the animal was inside the darkened goal box. Prior to surgeries, rats were trained and quickly acclimatized to the experimental conditions. For each rat, three independent trials were recorded.

Digital videos were recorded using a Nikon high definition Rebel T3 at a rate of 30 frames per second to capture data from underneath and the side simultaneously. Videos were analyzed in Windows Live Movie Maker, where still frames each were taken of animals in dorsiflexion and plantarflexion of the operated legs. To study any locomotor deficits, we analyzed the following gait parameters using ImageJ software (National Institutes of Health, Bethesda, MD, USA):

1. External paw rotation: During each foot placement on the injured and contralateral leg, the sagittal plane of the rat was drawn relative to foot placement, and angle of the paw at dorsiflexion was calculated in relation to the sagittal plane of the rat. External paw rotation was normalized to the control leg.
2. Dorsiflexion angle: To measure the dorsiflexion angle, the approximate location of the calcaneus and knee were determine and a line was drawn connecting those points of origin relative to the plane of the foot.
3. Plantarflexion angle: To measure plantarflexion angle, the approximate location of the paw in contact with the ground, calcaneus, and knee was determined. Lines connecting those three points were used to calculate the angle.
4. Hind limb spread: Hind limb spread was determined by first locating the sagittal plane of the rat. A midline was drawn on the rat to represent this plane. Middle of each hind limb paw was located and a line was drawn between them, perpendicular to the sagittal plane.
5. Stance to swing ratio: Movies were slowed to 0.5× frame rate, and the operated leg was observed for number of frames in and out of contact with the ground to calculate a stance/swing ratio.

### 2.4. Muscle Physiology 

Peak tetanic force, peak twitch force, percent recovery from sham, and force-frequency curves were measured. Rats were chosen at random and anesthetized using a vaporizer at 4% isoflurane/400 mL/minute O_2_. Following induction of general anesthesia, the sciatic nerve was isolated, and sural and peroneal branches ligated. Sciatic nerve was then stimulated using platinum electrodes connected to a Grass stimulator model SD9 (Astro-Med, Inc., Westwarwick, RI, USA) at 2-msec duration and 2-msec delay at varying voltages and frequencies. The knee and ankle joints were immobilized, and the Achilles tendon was cut from its insertion and connected to a MLT500/A force transducer (ADInstruments, Inc., Colorado Springs, CO, USA) with 2–0 silk sutures. Output was collected digitally using LabChart 8 software (ADInstruments). Optimal muscle length, stimulating voltage, and tetanic frequency were determined. During muscle lengthening, muscle was stimulated to tetanus for 3 s at each interval. Once optimal length was determined, tetanic contraction was stimulated at 3 s intervals until peak tetanic force dropped, indicating fatigue. Immediately following this force drop, three separate submaximal stimulations were measured. Those stimulations were used for muscle physiology assessments. Peak tetanic force was measured at maximum force for optimal frequency. Peak twitch force was measured at the peak of the submaximal force–time curve. Force–frequency curves were measured using maximum force for each respective frequency. Percent recovery was measured using maximum measured tetanic and twitch force in sham- and DMM-treated animals. These data were used to calculate the percent of functional recovery that DMM recovered compared to sham.

### 2.5. Histology

Whole gastrocnemius muscles were removed from operated and contralateral legs and fixed in 4% paraformaldehyde, dehydrated, and embedded in paraffin. Muscles were cross-sectioned approximately 0.5 cm from the margins. Sections (5 μm) were placed on Histobond slides (VWR, Radnor, PA, USA), deparaffinized and rehydrated, and stained with Harris’ haematoxylin (VWR) and eosin-Y (Thermo Scientific, Waltham, MA, USA) (H&E). Sections were also stained with Masson’s trichrome using Weighert’s haematoxylin (Sigma-Aldrich, St. Louis, MO, USA), Biebrich’s scarlet-acid fuschin (Sigma-Aldrich), and aniline blue (Sigma-Aldrich). Coverslips were mounted with xylene-based mounting media and allowed to dry flat before imaging.

### 2.6. Histomorphometry

Histomorphometry was used to quantify the percent of centrally located nuclei, myofiber diameter, ratio of muscle to collagen, and average blood vessel density present. Histological sections stained with haematoxylin and eosin (H&E) or Masson’s trichrome were imaged using a 10× and 40× objective, and were assessed as previously reported [25]. Healthy muscle from the sham-operated animals was used as a positive control. Histological analysis was performed on each muscle (n = 8) in three different locations within the graft area. Field sizes of 650 μm × 870 μm and 165 μm × 220 μm were used for histomorphometry. DMM locations were identified by first locating the zone of injury using injury margins. Once the zone was established, images were taken from three locations within the injury site, one at the margin, a second within the middle of the graft, and a third within an additional area of the graft away from the margins.

### 2.7. Immunohistochemistry 

Immunohistochemistry was used to stain for fetal myosin heavy chain (MyHC) paired box 7 (Pax7) nicotinic acetylcholine receptor-epsilon (AChR-ε), nicotinic acetylcholine receptor-gamma (AChR-γ), cluster differentiation-68 (CD68), cluster differentiation-163 (CD163), cluster differentiation-4 (CD4), cluster differentiation-8 (CD8), and FoxP3. Two AChR receptors were chosen to distinguish between mature adult acetylcholine receptors (AChR-ε) and fetal receptors (AChR-γ). Sections were deparaffinized and rehydrated prior to staining. To remove the methylated crosslinks from antibody-binding sites, slides were incubated at 95 °C for 5 min in 10 mM sodium citrate with 0.05% Tween-20. Slides were washed and then incubated with blocking serum (1.5% goat serum in PBS, Santa Cruz Biotechnology, Santa Cruz, CA, USA) for 1 h. Samples were washed with PBS/0.6% Tween-20, and incubated for one hour with primary antibody. Primary antibodies used in this experiment were: mouse anti-Pax7 (ab55494, Abcam, Cambridge, UK); rabbit anti-nicotinic acetylcholine receptor-epsilon (AChR-ε, ab65180, Abcam); mouse anti-nicotinic acetylcholine receptor-gamma (AChR-γ, MA3-043, Thermo Fisher Scientific, Waltham, MA, USA); mouse anti-myosin heavy chain-fetal (fMyHC, SC-53097, Santa Cruz Biotechnology); CD68 (ab125212, Abcam); CD163 (ab87099, Abcam); CD8 (MAB116, R&D Systems), CD4 (MAB554, R&D Systems), FoxP3 (MAB8214, R&D Systems) and were diluted in PBS with 1% BSA and 0.3% Tween-20. Samples were washed again with PBS/0.6% Tween-20 and incubated for one hour with secondary antibody diluted 1:200 in PBS/1% BSA/0.3% Tween-20. Secondary antibodies used in this experiment were from Invitrogen (Carlsbad, CA, USA): Alexa Fluor 594 (goat anti-mouse, A11005; goat anti-rabbit, A11012) and Alexa Fluor 488 (goat anti-mouse, A11001; goat anti-rabbit, A11008). Slides were washed three times for five minutes each with PBS, then incubated with DAPI (R37606, Invitrogen). Cover slips were mounted with anti-fade mounting media (Invitrogen) and imaged using a Zeiss laser confocal scanning microscope. For CD68, CD163, CD8, CD4, and FoxP3, infrared secondary antibodies were used (IRDye 700 and IRDye 800, LiCor) and imaged using a LiCor CLx scanner. To calculate the intensity/μm^2^, images were taken using a 63× oil immersion objective. Regions of interest were drawn carefully around individual muscle cells. Peripheral nuclei were included in sample, and thus some myocytes did overlap. Intensity within the region of interest was divided by the area of the region to obtain “intensity/μm^2^.” For samples that measured protein targets within nuclei (Pax7), regions of interest were drawn around the nuclei, and intensity/μm^2^ calculated. Quantification focused on DMM comparing RNU to Sprague Dawley to determine if treatment sites were different, while sham-stained sections were used only as staining controls and were not included in quantification.

### 2.8. Statistical Analysis 

Each variable was tested using N = 8 or N = 3 (empty defect) independent animals. The animal number was chosen based on a power analysis using an alpha of 0.05 and a power of 80% (delta = 5, sigma = 3, m = 1) to reveal a minimum of n = 7 per group to yield statistical significance. Data are presented as mean ± SEM with analysis done using GraphPad Prism 6.0 (GraphPad, La Jolla, CA, USA). Analysis comparing only two groups was performed by unpaired Student’s t-test, whereas analysis comparing more than two groups used one-way analysis of variance with Tukey’s post-hoc test. One way ANOVA with Tukey post-hoc test was used to determine if differences between rat strains existed, while an unpaired t-test was used to determine if VML-treated legs were different from sham controls. All *p* values < 0.05 were considered significant.

## 3. Results

### 3.1. Gait Analysis

**Figure 1:**
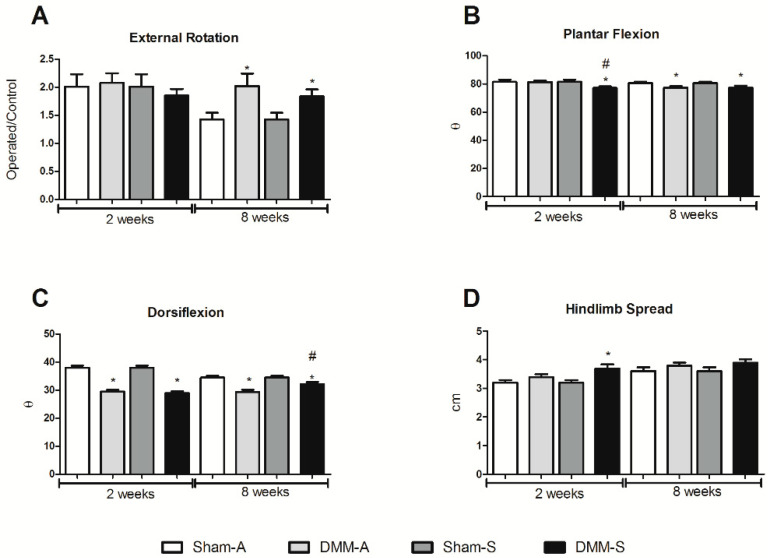
Animal gait demonstrated minimal differences between RNU (Sham-A and DMM-A) and Sprague Dawley (Sham-S and DMM-S) animals. Gait measurements demonstrated minimal changes in external foot rotation (**A**), a minor decrease in plantar flexion after 2 weeks (**B**), increased dorsi flexion after 8 weeks (**C**), and no change in hindlimb spread after 8 weeks (**D**). * Indicates a difference from sham control (*p* < 0.05, *t*-test) and # indicates a difference from RNU (*p* < 0.05, *t*-test). Data shown are means ± SEM of 8 animals.

Previous research indicated that a gastrocnemius VML wound caused an acute increase in external foot rotation, plantar flexion, and dorsiflexion [25]. When gait was assessed between Sprague Dawley and RNU rats, there was no change in external rotation at 2 weeks post injury, while external foot rotation remained elevated 8 weeks after injury in DMM-treated rats compared to sham controls (Figure 1A). At 2 weeks plantar flexion was significantly reduced in the Sprague Dawley rats compared to RNU rats, and DMM-treated rats showed lower plantar flexion angles compared to sham (Figure 1B). Dorsiflexion angles were reduced in all DMM-treated sites at weeks 2 and 8, and dorsiflexion increased at week 8 in the DMM-treated Sprague Dawley rats compared to RNU rats (Figure 1C), suggesting that gait was slightly altered in Sprague Dawley rats when only compared to RNU rats. In addition, hindlimb spread was unchanged between rat strains and higher in DMM-treated Sprague Dawley rats compared to sham at week 2, and no differences were measured at week 8 (Figure 1D).

### 3.2. Muscle Force

**Figure 2:**
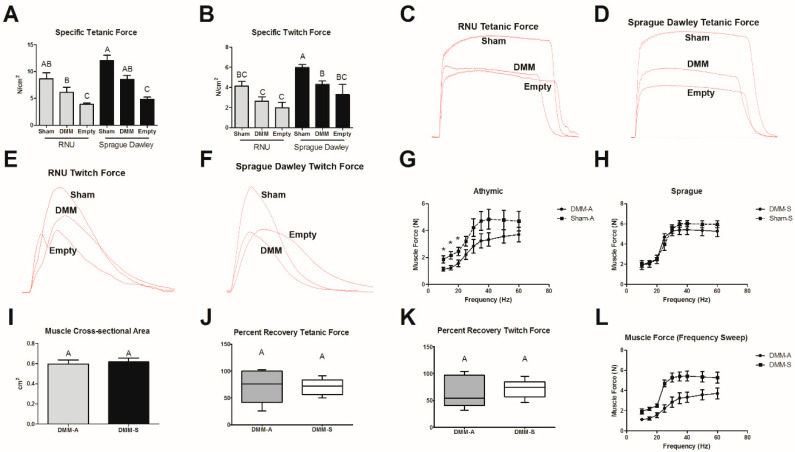
Comparison of muscle force output in RNU and Sprague Dawley rats treated with DMM. Specific tetanic force (**A**) and specific twitch force (**B**) both showed reduced muscle force when DMM-treated sites were compared to sham animals. Tetanic force (**C**,**D**) and twitch force (**E**,**F**) show representative force–time curves for each group tested. When animal groups were compared, Sprague Dawley rats produced more force than RNU rats. Force–frequency tests were used to further assess the physiologic differences between DMM and sham-operated animals for RNU (**G**), and Sprague Dawley (**H**) rats. Muscle cross-sectional area (**I**), percent recovery tetanic force (**J**), and percent recovery twitch force (**K**) were unchanged. Force–frequency of DMM-treated sites between RNU and Sprague Dawley rats were compared (**L**). * indicates a difference from sham in (**G**) (*p* < 0.05, *t*-test), Letters not shared indicate a significant difference (*p* < 0.05, Tukey). Data shown are means ± SEM of 8 DMM and sham animals and ± SEM of 3 for empty defect animals.

Tetanic and twitch force were measured in sham, DMM, and empty defect injury models to determine whether the VML model sufficiently reduced force output and if DMM treatments improved muscle force in a strain-dependent fashion. No differences were noted in tetanic force output between RNU and Sprague Dawley sham-treated, DMM-treated, and empty defect animals (Figure 2A). While tetanic force in both DMM-treated strains were not different from sham, empty defect animals were lower compared to sham and DMM. Specific twitch force was reduced between sham and DMM in Sprague Dawley rats but was unchanged in RNU rats. Twitch force generated by DMM-treated Sprague Dawley rats was higher than DMM-treated RNU rats (Figure 2B). Specific twitch force in empty defect animals was similar to DMM, but still lower than sham. In addition, there was a 32% reduction in tetanic force in DMM-treated Sprague Dawley rats and a 29% reduction in RNU rats. We also observed a 28% reduction in twitch force in DMM-treated Sprague Dawley rats and a 36% reduction in RNU rats. Representative force–time curves (C–F) demonstrate reduced maximum tetanic force for DMM and empty defect sites compared to sham.

Force–frequency was assessed in both Sprague Dawley and RNU rats to determine whether differences in stimulation frequency were detectable between sham and DMM animals (Figure 2G,H). Force was reduced in DMM-treated RNU rats at lower frequencies, but no differences were detected in Sprague Dawley rats. Furthermore, no differences in muscle cross-sectional area or percent recovery were detected between animal strain in DMM-treated sites (Figure 2I–K). A force–frequency test was also performed to determine whether differences were detectable in between RNU and Sprague Dawley rats. We demonstrated differences at lower frequencies and determined that our variability increased as frequency increased (Figure 2L).

### 3.3. Histological Assessment

**Figure 3:**
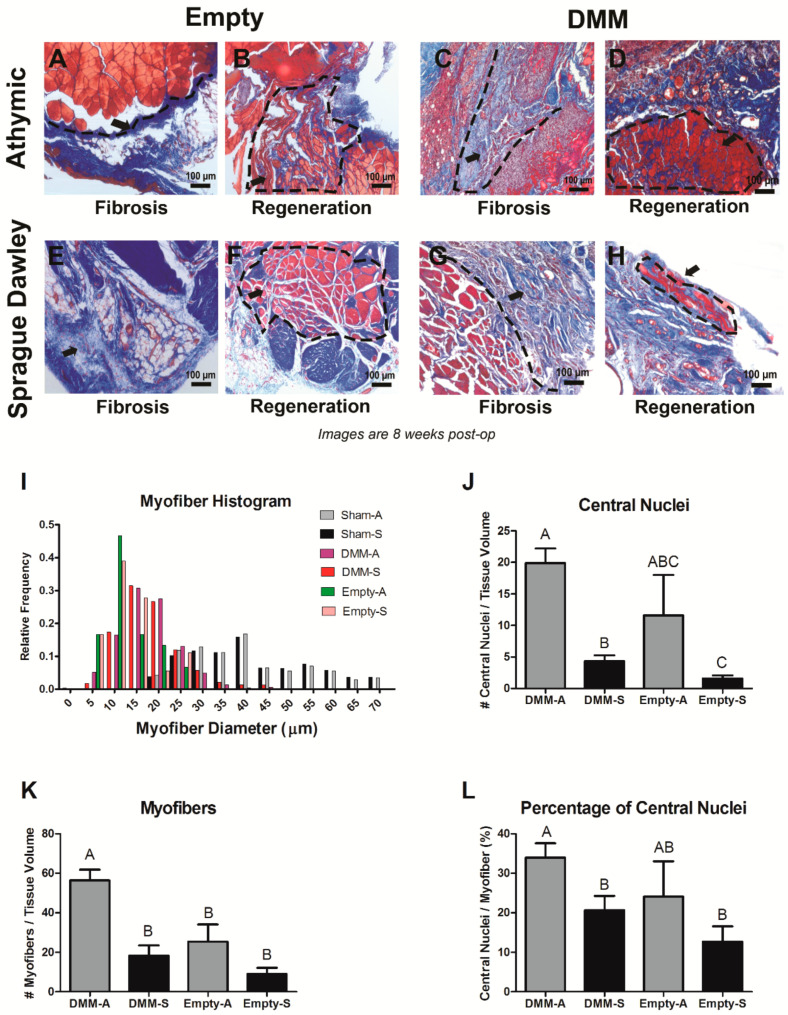
Histological staining and morphometric analysis for VML injuries in Sprague Dawley and RNU rats. Representative images demonstrate a fibrotic response following injury or DMM treatment (**A**,**C**,**E**,**G**), or regenerative response following injury or DMM treatment (**B**,**D**,**F**,**H**). Histomorphometry showed similar relative frequencies for myofibers in Sprague Dawley and RNU rats (**I**), increased central nuclei in DMM-treated RNU rats (**J**), increased myofibers in DMM-treated RNU rats (**K**), and increased percentage of central nuclei in RNU rats (**L**). Letters not shared indicate a significant difference (*p* < 0.05, Tukey). Data shown are means ± SEM of 8 DMM and sham animals and ± SEM of 3 for empty defect animals.

DMM-treated sites demonstrated different healing characteristics when RNU and Sprague Dawley rats were compared. Injury sites in RNU and Sprague Dawley rats appeared to respond differently between animals and strains. RNU empty defect injuries demonstrated a fibrotic and regenerative response (Figure 3A,B) as did DMM-treated sites (Figure 3C,D). This also occurred in empty defect and DMM-treated sites for Sprague Dawley rats, although the frequency and severity of fibrosis appeared to be greatest in Sprague Dawley compared to RNU (Figure 3E–H). We determined that RNU and Sprague Dawley rats had small muscle fibers (<20 μm) in the grafted area, and the relative frequency of small fibers was similar between RNU and Sprague Dawley rats (Figure 3I). Furthermore, empty defect sites appeared to have a higher relative frequency for small muscle fibers less than 15 μm. Whether this is related to less measurable fibers in the injury sites is unclear. Sham controls showed similar fiber size between strains. DMM-sites in both rat strains stained positively for centrally located nuclei (Figure 3J). In RNU rats, an average of 20 central nuclei fibers were identified per tissue volume, while only 5 fibers were identified in Sprague Dawley rats. Empty injury sites also showed a higher average number of centrally located nuclei per tissue volume in RNU rats compared to Sprague Dawley, although this was not significant. RNU rats showed no differences between empty defect and DMM-treated sites, while Sprague Dawley rats showed increased number of regenerated fibers in DMM-treated sites compared to empty defect. In addition, we showed there was an overall increase in the number of muscle fibers per tissue volume in RNU rats treated with DMM while empty sites for both animal strains were similar (Figure 3K). Centrally located nuclei per tissue volume correlated with the percentage of central nuclei fibers per myofibers measured, where 33% of fibers measured had centrally located nuclei. In contrast, Sprague Dawley-treated rats showed only 20% of fibers measured in a viewing area contained central nuclei (Figure 3L).

### 3.4. Immunohistochemistry

**Figure 4:**
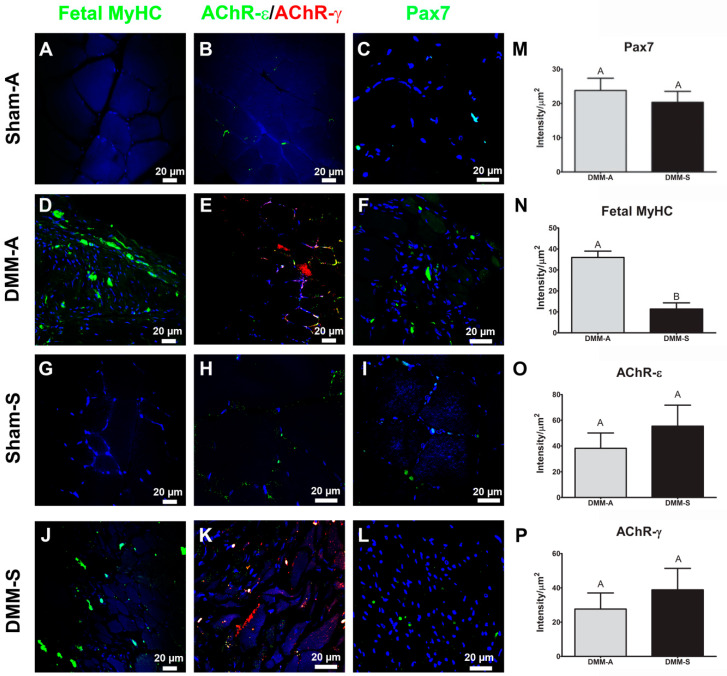
DMM supports satellite cells, de novo muscle fibers, and neuromuscular receptors in both Sprague Dawley and RNU rats. Immunostaining for fetal myosin heavy chain (fMyHC) (**A**,**D**,**G**,**J**), nicotinic acetylcholine receptor (AChR) -ε and -γ (**B**,**E**,**H**,**K**), and Pax7 (**C**,**F**,**I**,**L**) was quantified. Pax7 staining was similar (**M**), fetal MyHC was increased in RNU rats compared to Sprague Dawley (**N**), AChR-ε was unchanged (**O**), and AChR-γ was unchanged (**P**). Representative stained sections for RNU (**E**) and Sprague Dawley (**F**) rats are shown. Data shown are representative images from 8 animals and are means ± SEM of 8 animals. Letters not shared indicated a significant difference (*p* < 0.05, Tukey).

**Figure 5:**
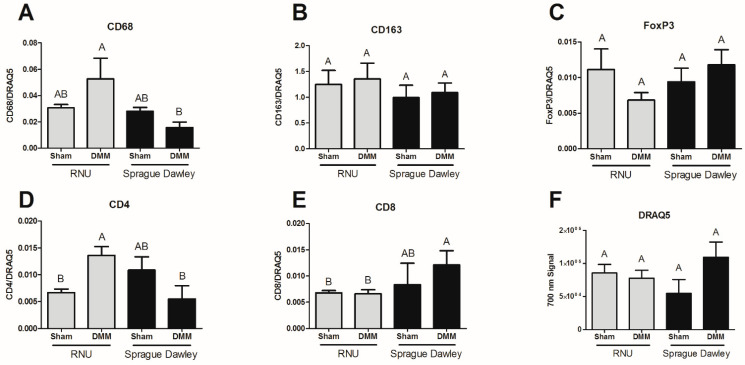
CD68+ staining is increased in RNU DMM-treated sites compared to Sprague Dawley. CD68 staining was quantified and showed increased levels in RNU rats (**A**). Yet, CD163 (**B**) and FoxP3 (**C**) were unaltered. CD4 staining was increased in RNU rats (**D**) while CD8 staining was lower (**E**). In addition, CD4, CD8, and FoxP3 levels were low. DRAQ5 showed no change in DNA staining (**F**). Data shown are means ± SEM of 8 animals. Letters not shared indicated a significant difference (*p* < 0.05, Tukey).

Pax7, fetal MyHC, AChR-ε, and AChR-γ stained sections demonstrated similar results among all tested antibodies except for fetal MyHC. Graft areas stained positively for Pax7, fetal MyHC, AChR-γ, and AChR-ε (Figure 4A–L) with sham acting as staining controls. Stained sections were quantified only in the DMM-treated sites using staining intensity normalized to cell area. No differences were detected between RNUs and Sprague Dawley DMM-treated sites for Pax7, AChR-γ, and AChR-ε. In contrast, fetal MyHC was elevated in RNU-treated rats versus Sprague Dawley-treated rats (Figure 5A–D). Representative images of RNU (Figure 4E) and Sprague Dawley (Figure 4F) are shown.

### 3.5. Immune Cell Staining

Tissue sections were stained using antibodies against CD68 (phagocytic macrophages), CD163 (non-phagocytic macrophages), CD4 (naïve T cells), CD8 (cytotoxic T cells), and FoxP3 (T regulatory cells). CD68 was reduced in Sprague Dawley DMM-treated sites versus RNUs (Figure 5A), while CD163 showed no statistical differences (Figure 5B). Moreover, CD68 staining in DMM-treated sites was similar to sham controls. FoxP3 stained sections were not different between RNU and Sprague Dawley DMM-treated sites and were similar to baseline sham controls (Figure 5C). In addition, we quantified CD4 (Figure 5D) and CD8 (Figure 5E) and determined that CD4 levels were higher in RNU rats and CD8 levels were higher in Sprague Dawley rats treated with DMM compared to RNU, but were not different from sham. DRAQ5 DNA staining was unchanged between strains and treatments (Figure 5F).

## 4. Discussion

In this study, we tested the DMM graft performance in two commonly used animal strains in regenerative medicine. We demonstrated that DMM supported muscle fiber regeneration in Sprague Dawley and RNU rats, and that twitch force was greatest in Sprague Dawley rats compared to RNU. We showed that tetanic force output was reduced in empty defect models compared to sham, indicating that our model sufficiently caused muscle deficits. To further determine muscle force properties and test the hypothesis that DMM would improve histologic and functional evidence of muscle regeneration in RNU rats when compared to immunocompetent Sprague Dawley rats, we tested the muscle force output over a series of frequencies and found no differences between sham and DMM-treated RNU rats at higher frequencies (25–60 Hz), while DMM-treated sites in Sprague Dawley rats were unchanged compared to sham at all frequencies. Together, muscle force data suggested strain-dependent differences in force production, but were unable to identify strain-dependent differences in muscle regeneration. We then assessed histology and showed differences in muscle fiber formation, where RNU rats were far better equipped to form muscle fibers in the graft area compared to Sprague Dawley. We next wanted to determine whether those differences could be ascribed to changes in satellite cells (Pax7), synapse formation (AChR), or newly formed muscle fibers. Indeed, we only demonstrated differences in newly formed muscle fibers (fetal MyHC). We hypothesized that immune cell composition might explain the increased muscle fiber formation in RNU rats. Interestingly, RNU rats stained more intensely for CD68 (phagocytic) macrophages and CD4 helper T cells while CD8 (cytotoxic) T cells staining intensity was higher in Sprague Dawley rats. Overall, our data raise awareness that strain-dependent differences in muscle regeneration and functional recovery exist between Sprague Dawley and RNU rats, and that regenerative medicine methods to test for fiber formation and muscle function need to take the type of animal model used into consideration.

Biologic materials developed for VML testing have shown promise using various animal models. Among these models, Sprague Dawley and RNU rats are more commonly used in regenerative medicine tests. Sprague Dawley rats are an outbred strain of rat making their genetic composition more unique. In contrast, RNU rats are an inbred strain developed solely for the purpose of testing allogeneic and xenogeneic transplants. Regenerative medicine has focused on developing products that combine allogeneic or xenogeneic cells and biologic materials. Often, cells and biologics are derived from a xenogeneic source, and this is especially the case when developing human-based materials. Human materials are considered the “gold standard.” Yet, it is difficult to test their efficacy without using an immunocompromised animal such as an RNU rat, presenting a special challenge when attempting to interpret data following surgical repair in rats that are predisposed to improved healing characteristics.

While forkhead box proteins (*Fox*) are well studied, the relationship between *Foxn1*-deficiency in nude rodents and improved regeneration is not well understood. *Foxn1* is a transcription factor that is predominantly expressed in skin cells, and it is hypothesized that *Foxn1* regulates the switch between scar-forming and scar-free healing [26]. In mammals, the scar-forming process occurs in three overlapping phases during wound—inflammation, new tissue formation, remodeling—and these same phases apply to skeletal muscle. Interestingly, other studies also showed that nude mice and rats (*PrkdcSCID/SzJ* and *Rag1tm1Mom*) possess the inherent ability to regenerate tissue with reduced scar, while thymectomy did not alter the scar-forming response [9,11,12]. This supports the idea that genetically deficient, nude rodents regenerate differently than their immunocompetent counterparts.

In skeletal muscle, differences between RNU and wild type mice were investigated using VML injury models [29]. Corona et al. focused on the development of a VML injury in the tibialis anterior muscle treated with minced autololgous muscle. In particular, they determined that no differences in total fiber number were observed and no differences in response to treatment were observed; however, treatments originated from autologous tissue and thus it is unclear whether differences in repair and regeneration could be observed. Interestingly, representative histological sections appear to show different levels of cell infiltrate, but these results were not characterized in the manuscript. Our study differs from Corona et al. We tested decellularized muscle allografts and not minced autologous grafts, and our model used the gastrocnemius instead of the TA. TA and gastrocnemius muscles were reported to respond differently in a nerve injury model, suggesting these muscles have different healing responses [30].

Our study is limited in the regard that we did not specifically test wild type versus *Foxn1*-deficient animals to gauge the true effect of *Foxn1* deficiency on skeletal muscle regeneration; however, our data do indicate that commonly used animal models in regenerative medicine should be carefully considered when assessing regenerative data. For instance, force generation in *Foxn1*-deficient rats was less on average compared to Sprague Dawley rats. Morrison et al. determined that *Foxn1* −/− mice had smaller muscle fibers compared to WT controls, indicating reduced force output [13]. Moreover, while force recovery 8 weeks post-injury was unchanged in RNU rats compared to Sprague Dawley, 38% of RNU rats had greater than 85% recovery and only 12% of Sprague Dawley rats showed a high degree of recovery. These data suggest that RNU rats responded better to DMM treatment than Sprague Dawley rats. When muscle force and histology are compared, it was evident that fibrosis within the treated areas contributed mostly to muscle force output and not de novo fiber formation. It is possible that RNU wound healing and repair improved force transmission from the intact proximal site to the distal injury site in a way that was different from Sprague Dawley, but more study is needed to fully determine this.

Data collected in this study also support the idea that RNU rats provide a type of microenvironment that supports more muscle fiber growth compared to Sprague Dawley rats. This is exemplified by the number of small diameter muscle fibers, more centrally located nuclei, and increased staining for fetal MyHC. In addition, empty defect sites in RNU rats appeared to have an improved capacity to regenerate muscle fibers following injury compared to Sprague Dawley rats as shown by centrally located nuclei, providing more supportive evidence that RNU rats heal differently. This is significant because muscle fiber regeneration in mild injuries occurs within 28 days after injury, and VML injuries frequently lack de novo fiber formation 8 weeks after injury without the addition of stem cells [31]. It is possible that mature muscle fibers (MyHC I or II) were also present in injury sites at 8 weeks in addition to fetal MyHC, but our focus was to test the whether *Foxn1* deletion affected fetal MyHC staining.

Previous research demonstrated a genetic linkage between *Foxn1* and Myh3 [4]. We observed increased fetal MyHC staining, which is driven by Myh3 RNA, but whether this linked to increased Myh3 transcription in RNU rats due to their *Foxn1* deficiency was not tested. Interestingly, we did not observe a concomitant increase in Pax7 staining. This could be due to a subsequent decrease in the number of Pax7+ cells present after 8 weeks [32,33], or this indicates that the environment produced by RNU rats does not affect Pax7. In addition, AChR gamma levels were unchanged between rat strains. More study is needed to determine whether satellite cells numbers and AChR distribution are altered between the two types of rats.

The inflammatory response plays a critical role in muscle regeneration after injury. Of the major inflammatory mediators, macrophage participation is considered critical and highly regulated. In addition, the adaptive immune response is gaining momentum as a critical regulator of tissue regeneration [34]. Interplay between T cells and macrophages has been investigated in musculoskeletal tissues [21] and as a function of the host response to a biomaterial [21,35]. It is widely accepted that T-cells activation is dependent on macrophage interaction, and this communication plays an important role in wound healing and cancer, but how this is affected in nude rodents is not clear. While there are little data describing the effect of nude animals on macrophage function, one report suggests that nude animals alter the functional state of microglia/macrophages in the brain based on a change in morphological shape and reduced inflammatory mRNA levels [36]. One of the limitations of our model is that we do not stain for immune cell infiltrate at earlier time points. This limits our ability to interpret results since acute inflammation has mostly resolved. Our data demonstrated elevated levels of CD68 (M1) macrophages in RNU DMM-treated sites after 8 weeks compared to Sprague Dawley-treated sites. In contrast, overall levels of CD163 (M2) macrophages were higher than CD68, but Sprague Dawley and RNU injury sites were similar. Precise markers for M1 and M2 are difficult considering that both phenotypes can express each marker during repair. While CD68 has been previously identified as an M1 macrophage marker, it also was used as a way to identify all macrophages, which could also limit interpretation of our results. Furthermore, Pan et al. also showed that RNU mice were associated with reduced chemokine ligand 5 (CCL5) levels, a chemoattractant produced by microglia and macrophages that plays a significant role in wound healing and CD-8+ T-cell recruitment [37]. Our study demonstrated reduced levels of CD8 staining in RNU DMM-treated rats compared to Sprague Dawley DMM-treated rats. These data support prior studies that also found reduced CD-8 levels in nude mice [36], although Sprague Dawley sham levels were not different from DMM-treated rats. Moreover, our data showed that CD-4 T cells were increased in RNU rats further suggesting that T cell and macrophage communication was altered in RNU rats. While these data are purely correlative and are limited to very specific cellular markers, it could help explain why nude rats show enhanced muscle regeneration. More study is needed to investigate how inflammation plays a role in material responses to immunocompromised animals.

## 5. Conclusions

In both animal types, DMM supported the formation of myofibers. However, regeneration was markedly less in the Sprague Dawley animals when compared to RNU. Interestingly, we observed an increase in the proportion of M1 macrophages in the RNU animals with no change in M2. We also observed an increased proportion of helper T cells and reduced cytotoxic T cells in RNU rats compared to Sprague Dawley rats. Several aspects of this study need more examination, including how specific biomaterials regulate macrophage and T-cell communication and whether loss of this communication is indeed responsible for the observed results, or if those results were specifically due to the loss of *Foxn1*. RNU VML rat models are valuable in cases where regenerative strategies that use xenogeneic components are used. However, our study indicates that results should be interpreted with caution since the T-cell deficient state of these rodents appears to promote skeletal muscle regeneration.

## Data Availability

The data presented in this study are openly available in https://figshare.com/.
